# A Case of Posterior Interosseous Nerve Palsy Associated With Robotic Low Anterior Resection

**DOI:** 10.7759/cureus.36170

**Published:** 2023-03-15

**Authors:** Ryo Ohno, Yoshiko Matsumoto, Kurumi Sahara, Ryuji Kajitani, Toshifumi Watanabe, Gumpei Yoshimatsu, Shinsuke Fujioka, Hiromu Ogura, Yoshio Tsuboi, Suguru Hasegawa

**Affiliations:** 1 Department of Gastroenterological Surgery, Fukuoka University Hospital, Fukuoka, JPN; 2 Department of Neurology, Fukuoka University Hospital, Fukuoka, JPN; 3 Department of Neurology, Fukuseikai Hospital, Fukuoka, JPN

**Keywords:** complication, peripheral motor neuropathy, early postoperative complication, robotic colorectal surgery, posterior interosseous nerve palsy

## Abstract

A peripheral nerve compression injury associated with surgical positioning is an important complication that might compromise quality of life. We report a rare case of posterior interosseous nerve (PIN) palsy after robotic rectal cancer surgery. A 79-year-old male with rectal cancer underwent robotic low anterior resection in a modified lithotomy position with both arms tucked at his sides with bed sheets. Following surgery, he felt difficulty moving his right wrist and fingers. A neurological examination revealed muscle weakness in the area innervated by the PIN alone without sensory disturbance, and he was diagnosed with PIN palsy. The symptoms improved with conservative treatment in about a month. The PIN is a branch of the radial nerve and controls dorsiflexion of the fingers, and intraoperative continuous pressure on the upper arm by right lateral rotation position or by the robot arm was considered to be the cause.

## Introduction

Peripheral nerve injury is a potential complication that can occur during surgery and might compromise quality of life. The most common mechanisms of peripheral nerve injury during surgery are mechanical compression, stretch, or direct trauma to the nerves, which are associated with patient positioning. In the arm, the nerves that are most commonly affected by compression during surgery are the brachial plexus and ulnar nerves, which originate from the spinal cord in the neck and extend down the arm. Compression of these nerves can result in symptoms such as weakness, numbness, or tingling in the arm or hand and may impact the patient's ability to move the affected limb. The posterior interosseous nerve is a branch of the radial nerve and provides motor innervation to the muscles of the forearm that extend the wrist and fingers. We report a rare case of posterior interosseous nerve palsy after robotic rectal cancer surgery.

## Case presentation

**Figure 1:**
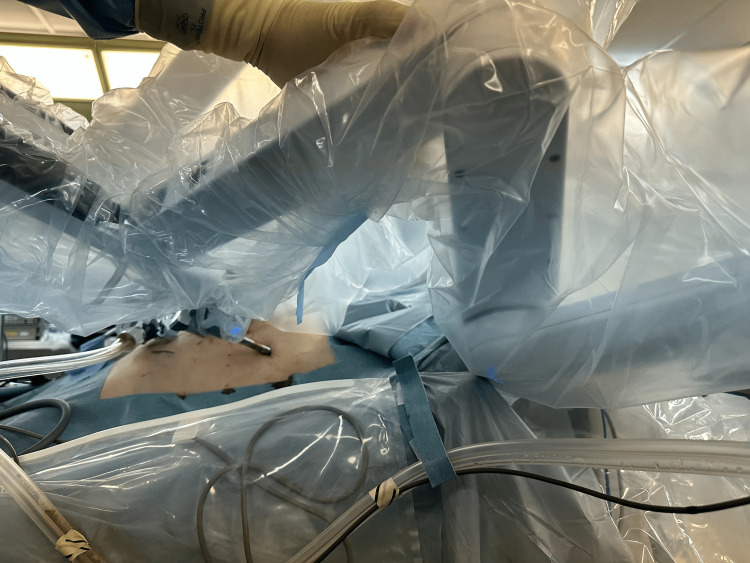
Contact between the fourth arm and right upper extremity It was recognized that the fourth arm occasionally came into contact with the left arm area during surgery, but due to the surgical drape, it was not possible to determine the actual degree of pressure being applied.

The patient was a 79-year-old man diagnosed with rectal adenocarcinoma (cT1bN0M0). His height was 163 cm, and his weight was 47.3 kg, giving him a body mass index of 17.3. He has no significant past medical history, such as diabetes or hypertension. He underwent robotic low anterior resection with diverting ileostomy under general and epidural anesthesia, with an arterial line placed in the left arm. He was placed in a modified lithotomy position with both arms tucked at his sides with bed sheets and the arm positions secured with sleds. The robotic procedure was performed with the patient positioned in a 15-degree Trendelenburg and 5-degree right lateral rotation for 210 minutes. The total operation time was 6 h 38 mins with estimated bleeding of 5 mL. It was recognized that the fourth arm occasionally came into contact with the left arm area during surgery, but due to the surgical drape, it was not possible to determine the actual degree of pressure being applied (Figure 1).

On postoperative day one, he was noted to have difficulty moving his right wrist and fingers. The clinical finding was wrist drop, which was characterized by impaired dorsiflexion of the wrist and thought to be associated with some kind of radial nerve palsy. Manual muscle testing (MMT) revealed muscle weakness in the area innervated by the posterior interosseous nerve but normal muscle strength in other areas innervated by the radial nerve as well as the median and ulnar nerves (Table 1). A sensory disturbance was not observed. A motor nerve conduction study using the inching technique revealed reduced amplitude between 65 mm and 75 mm proximal from the ulnar styloid process, suggesting posterior interosseous neuropathy (Table 2). However, there were no abnormal findings on magnetic resonance imaging, such as a fracture, hematoma, or a mass in the right upper extremity. Finally, he was diagnosed as having right posterior interosseous nerve palsy, which was treated conservatively, improved gradually, and recovered completely by one month following surgery.

**Table 1:** Manual muscle test (MMT) results

Muscle (right arm)	MMT
Extensor carpi radialis	5
Triceps brachii	5
Supinator	5
Extensor carpi ulnaris	4
Extensor digitorum	2
Extensor digiti minimi	2
Abductor pollicis longus	3
Extensor hallucis	3
Extensor indicis	3

**Table 2:** Results of motor nerve conduction tests on the right posterior interosseous nerve using the inching technique

Point	A	B	C	D	E
Distance* (mm)	25	35	45	55	65
Latency (ms)	2.2	2.5	2.6	2.8	3
Amplitude (mV)	3.6	2.8	2.6	2.5	2.3
Area (mVms)	21.9	21.6	21.6	21.5	20.5
*Distance 4 cm proximal from the ulnar styloid process.					

## Discussion

Although SSI is the most common postoperative complication after colorectal surgery, intraoperative peripheral nerve injuries are also serious complications that cause pain and suffering to patients. In addition, these complications have been associated with negative economic impact, increased morbidity, an extended postoperative hospital stay, readmission, sepsis, and death [1,2]. Most of these injuries occur in the pelvic region and are associated with the positioning of the lower limbs during lithotomy [3]. With regard to the upper extremity, injuries to the brachial plexus and the ulnar and radial nerves due to intraoperative compression are common. To our knowledge, this is the first report of posterior interosseous nerve palsy occurring after robotic colorectal surgery.

The posterior interosseous nerve is a deep branch of the radial nerve, which originates from the C7 and C8 segments in the cervical spine and innervates the extensor compartment of the forearm. The characteristic feature of this nerve disorder is motor impairment, which manifests as a wrist drop in an area without sensory disturbance. The diagnosis is relatively easy based on the characteristic symptoms [4], but evoked electromyography is useful for a definitive diagnosis. The most common cause of this neuropathy is persistent compression of the nerve by the arcade of Frohse [5], a ganglion [6,7], or a lipoma [8]. However, no such findings were observed in the present case, and persistent intraoperative compression on the right upper arm from the low right position or compression by a robotic arm was considered to be a potential cause.

Prevention of nerve compression during surgery is key to avoiding intraoperative peripheral nerve palsy. The instruction manual for da Vinci states to be careful about contact between the arm and the patient. However, the upper body is draped during surgery, so the pressure on the forearms from the body side plates or a robotic arm may not be noticed (Figure 1). In our facility, after the occurrence of neurological complications due to compression, we perform visual inspections and pressure monitoring of the patient's limbs every 1-2 hours during surgery. We feel that continuous pressure monitoring applied to limbs during surgery is effective because it can detect excessive pressure even if the limbs are not directly visible due to drapes, and corrections can be made through limb positioning adjustments [9]. In order to prevent this type of positioning-related peripheral neuropathy, we believe it is important to use appropriate positioning and to check regularly for pressure on any part of the body during surgery.

## Conclusions

We report a rare case of posterior interosseous nerve palsy after robotic rectal cancer surgery. Prevention is key to avoiding intraoperative peripheral nerve palsy. Surgeons should minimize the risk of nerve injury during surgery, including through careful patient positioning and avoiding prolonged pressure on the nerves.
